# Should we recommend spaying cats for mammary tumour prevention, and if so, at what age?

**DOI:** 10.18849/ve.v10i3.711

**Published:** 2025-08-15

**Authors:** Elena Gogua+

**Keywords:** CATS, FELINE, MAMMARY TUMOUR, NEUTERING, OVARIOHYSTERECTOMY, RISK, SPAYING

## Abstract

**PICO question:**

In cats does spaying versus non-spaying reduce the risk of mammary tumours?

**Clinical bottom line:**

**Category of research:**

Risk.

**Number and type of study designs reviewed:**

Four studies were reviewed: three case-control studies and one retrospective cohort study.

**Strength of evidence:**

Weak.

**Outcomes reported:**

All four studies found a protective effect of spaying female cats against mammary tumour. One study assessed the age of spaying and found a protective effect only when spaying was performed before 1 year of age, whereas spaying after 2 years of age was associated with an increased risk of developing mammary tumour compared to intact cats. These publications contain a moderate to high risk of bias.

**Conclusion:**

Based on the available evidence, spaying appears to reduce the risk of mammary tumours development in female cats. The data suggest that the protective effect is more pronounced when spaying is performed at an earlier age. However, due to the weak nature of the current evidence, further well-designed clinical trials are needed to confirm these findings and determine the optimal age for spaying.

## How to apply this evidence in practice

The application of evidence into practice should take into account multiple factors, not limited to: individual clinical expertise, patient’s circumstances and owners’ values, country, location or clinic where you work, the individual case in front of you, the availability of therapies and resources.

Knowledge Summaries are a resource to help reinforce or inform decision-making. They do not override the responsibility or judgement of the practitioner to do what is best for the animal in their care.

## Clinical scenario

An owner brings in a 3-month-old kitten for vaccination. They have read in a magazine that early spaying protects female cats from mammary tumours. They ask the veterinarian: 'Is this true? And if so, at what age should the operation be performed?'

## The evidence

Four primary research studies met the PICO question, investigating the influence of spaying on the risk of mammary tumours in cats – three case-control studies (Dorn et al., 1968, Overley et al., 2005, Graf et al., 2016) and one cohort study (Hayes et al., 1981). Additionally, there is a case-control study (Misdorp et al., 1991) for which the full text was not accessible at the time of this Knowledge Summary’s literature search. The available studies are retrospective and provide weak evidence for a protective effect of spaying on the risk of mammary tumours in cats.

## Summary of the evidence

**Dorn et al. (1968):** Survey of Animal Neoplasms in Alameda and Contra Costa Counties, California. II. Cancer Morbidity in Dogs and Cats from Alameda County **Aim:** To investigate the natural history of cancer in pet dogs and cats in a defined geographic area and to provide histologically confirmed cases for epidemiological analysis.

Population:	Cats with malignant mammary tumours, reported in the central animal neoplasm registry from Alameda County, California, during 1963–1966 (3 years).
Sample size:	21 cats.
Intervention details:	The total number of tumour cases (256) were categorised by their primary site according to the Manual of the International Statistical Classification of Diseases, Injuries, and Causes of Death to obtain the number of mammary tumours (21 cases). The baseline animal population data (control group) were collected from a probability sample survey of households in Alameda County conducted by the Human Population Laboratory in 1965, which included 706 cats. Reported cases and baseline population data from Alameda County were used to measure the effect of sex and breed on the risk of developing cancer of specific sites. For tests of association regarding mammary gland cancer, spayed (vs. intact) was controlled, except when spaying was the test factor.
Study design:	Case-control study.
Outcome Studied:	The relative risk of developing a mammary tumour if spayed. Annual incidence rates per 100,000 for spayed and entire cats. The effect of sex on the risk of developing cancer of specific sites.
Main Findings (relevant to PICO question):	The intact female cats had approximately a sevenfold higher relative risk of mammary cancer than neutered females (Relative Risk for spayed cats 0.15, P = 0.0037). The annual incidence rates of mammary tumour were 20.4 per 100,000 for spayed cats and 31.8 per 100,000 for entire cats.
Limitations:	A small sample size of cats. Data on spaying of cats were collected by interviewing owners, so there is a risk of recall bias. No data was provided on the spaying status of the 21 cats with mammary tumours. The control group was a population of cats collected as part of a probability sample survey of households. It is uncertain whether animals in this group had mammary tumours. It is unclear how many of the surveyed cats were used as controls in the statistical analysis. The criteria for diagnosing mammary tumours are unclear. Lack of clarity about statistical analysis.

**Graf et al. (2016):** Swiss Feline Cancer Registry 1965–2008: the Influence of Sex, Breed and Age on Tumour Types and Tumour Locations **Aim:** To analyse the influence of sex, neutering status, breed, and age on the development of the most common feline tumours types and tumour locations using data from the Swiss Feline Cancer Registry.

Population:	Cats with confirmed tumours submitted to the Swiss Feline Cancer Registry between 1965 and 2008 (43 years). Registry data were compiled from three diagnostic laboratories across Switzerland. A total of 41045 cats (male and female) were included in the registry. The appraisal focuses on female cats only.
Sample size:	23216 female cats: 1501 cases (cats with mammary tumours) 21715 controls (cats with other tumours).
Intervention details:	The registry provided information on sex, spaying status, breed, and age on the development of tumours of various locations. No baseline population was used; instead, the study compared cases of cats with mammary tumours to those without, using other cases in the database. This information was then included in the multiple logistic regression model. The analysis used multiple logistic regression adjusting for region, age, breed, year, and method of examination. In line with the PICO question, only details regarding the effect of spaying on mammary tumours were included in the evidence assessment.
Study design:	Case-control study.
Outcome Studied:	Risk of developing a mammary tumour between spayed vs. entire cats.
Main Findings (relevant to PICO question):	The odds of spayed female cats developing a tumour in the mammary gland compared with entire female cats were lower, indicating a reduced risk associated with spaying: for all tumours mammary gland OR 0.62, 95% CI 0.55–0.7, P < 0.01 for malignant tumours mammary gland OR 0.69, 95% CI 0.61–0.79, P < 0.01.
Limitations:	Data were collected over several decades, which may introduce biases related to changes in diagnostic criteria, record-keeping practices, and population demographics over time. The problem of multiple comparisons was not considered. Only cats from the cancer registry were included in the study, which introduces a selection bias toward animals already diagnosed with tumours, rather than a general at-risk population. There are no data on the number of spayed and intact female cats, only the results of a statistical analysis expressed in OR (95% CI).

**Hayes et al. (1981):** Epidemiological features of feline mammary carcinoma **Aim:** To examine the epidemiological characteristics of mammary carcinoma in domestic cats from a well-defined population across 15 veterinary teaching hospitals in North America.

Population:	Domestic cats from clinical visits at 15 North American veterinary medical teaching hospitals between 1964 and 1978 (14 years). The Veterinary Medical Data Program (VMDP) collected data on 132 cats with microscopically confirmed primary mammary tumours of various histological types. Of the 132 cats with mammary tumours, 21 with non-carcinoma types were excluded. One cat was excluded due to unknown spay status. The analysis included only 110 female cats with carcinoma relevant to this PICO.
Sample size:	110 female cats with mammary carcinoma: 58 intact cats 52 spayed cats.
Intervention details:	For statistical comparison, the at risk population was expressed in the epidemiological measure of “cat-years”: each cat was counted once for every calendar year in which it visited the clinic at least once for any reason, across the study period (1964–1978). The independent effects of various zoographic factors on the case series were evaluated by the relative risk (RR). Cases were tabulated by tumour cell-type and malignancy behaviour, breed, sex and age at first confirmed diagnosis.
Study design:	Retrospective cohort study.
Outcome Studied:	The relative risk of developing mammary carcinoma in female cats, comparing spayed to intact individuals, was analysed.
Main Findings (relevant to PICO question):	The relative risk in spayed females was significantly less than intact females (RR = 0.6, 99% CI = 0.33–0.94), adjusting for variations in age and breed. Surgical data were available for 15/of the 52 spayed cats with mammary carcinoma. Four of these cats had been spayed before the age of two years; the mean time lapse between oophorectomy and carcinoma development was 54 months.
Limitations:	There are only RR results. There is no information about what indicators were used for statistical analysis. Only cats with mammary carcinoma were included in the study, which introduces a selection bias toward sick animals. The disproportionate large number of Siamese cats in the sample (n = 52/110) may bias the results, because it does not reflect the general at-risk population. It is unclear how cases were selected for the study.

**Overley et al. (2005):** Association between Ovarihysterectomy and Feline Mammary Carcinoma **Aim: **To assess the effects of age at ovariohysterectomy, parity, and progestin exposure on the risk of feline mammary carcinoma development.

Population:	Female cats with histopathological diagnosis mammary carcinoma (cases), and with conditions other than mammary lesions (controls) by the outpatient biopsy service at the Matthew J. Ryan Veterinary Hospital of the University of Pennsylvania from 2000 to 2001 (1 year).
Sample size:	404 female cats: 204 cases 200 controls.
Intervention details:	Controls were selected to have a similar distribution of age and year of diagnosis as the cases. Information about the patient’s sex, age, breed, exogenous progestin exposure, ovariohysterectomy status, age at ovariohysterectomy, parity, number of litters, and tumour type were obtained through biopsy reports, questionnaires sent to referring veterinarians, phone interviews with owners, and review of medical records. Cats were grouped according to age at spaying: up to 6 months, 6–12 months, 13–24 months, over 2 years, and intact.
Study design:	Case-control study.
Outcome Studied:	The odds ratios to assess the relationship between development of mammary carcinoma and spayed status in cats. The effect of age of spaying on risk of feline mammary carcinoma development.
Main Findings (relevant to PICO question):	Intact female cats represented n = 41/150 (27%) of cases and n = 17/131 (13%) of controls. Intact female cats were at increased risk of having mammary carcinoma (OR 2.7, 95% CI 1.4–5.3, P < 0.001). Of those that were spayed, the age of ovariohysterectomy was known for 109 cases and 114 controls (total N = 223). Female cats had a 91% reduction in the risk of developing a feline mammary carcinoma if spayed prior to 6 months of age (OR 0.09, 95% CI 0.03–0.24) and an 86% reduction in risk if spayed prior to 1 year of age (OR 0.14, 95% CI 0.06–0.34) compared with intact female cats. Data also indicated a small and statistically non-significant risk reduction of 11% (OR 0.89, 95% CI 0.35–2.3) if female cats were spayed between 13 and 24 months of age, compared with intact females. Ovariohysterectomy performed after two years of age increased the risk of feline mammary carcinoma development when compared with intact female cats (OR 3.7, 95% CI 1.3–12.5).
Limitations:	Spaying age was not known for all cats. The reproductive history data were obtained from questionnaires sent to referral veterinarians, so there is a risk of bias due to potential subjectivity of responses, confusion, and inadequate record keeping. The control population included female cats diagnosed with tumours of organs other than the mammary gland. Since these were not healthy animals, this could be a source of bias. The problem of multiple comparisons was not considered.

## Appraisal, application and reflection

Spaying female cats is one of the most common veterinary procedures. There is a widespread recommendation among veterinarians to perform it before the first oestrus for the greatest reduction in the risk of mammary tumours. However, there are very few studies that extensively investigate this issue.

The search found four studies (Dorn et al., 1968, Hayes et al., 1981, Overley at al., 2005, Graf et al., 2016); they were all retrospective analysis of medical records, which limits the control of confounders, and one case-control study (Misdorp et al., 1991) for which the full text is not available.

The first study was published more than fifty years ago (Dorn et al., 1968). The authors used population data from Alameda County, California, to calculate estimated cancer incidence rates in dogs and cats and measure the effects of sex and breed on site-specific cancer risk. Regarding the PICO question, the authors of the original study concluded that intact cats had a relative risk of mammary tumours seven times higher than spayed cats (relative risk (RR) for spayed cats = 0.15). The study has many limitations: relatively few cats with mammary tumours, population data were collected as part of a probability sample survey of households, population rates were calculated using unclear coefficients, lack of clarity about statistical analysis. Therefore, the strength of evidence is weak.

Hayes et al. (1981), reported similar findings. This epidemiological study assessed the influence of breed, age, sex, and gonadal status on the risk of developing mammary tumours. The study utilised data collected from 15 veterinary teaching hospitals in North America through the Veterinary Medical Database Program (VMDP) from March 1964 to June 1978. A total of 132 cats with mammary tumours were identified. Of these 132 cats, only 111 with carcinomas were included in the analyses of breed, age, and gonadal status; one of these had an undetermined gonadal status, leaving 110 cats in the final comparison by spay status. Relative risk (RR) was evaluated considering age and breed. Regarding the PICO question, spayed cats had a significantly lower risk of developing mammary carcinoma compared to non-spayed cats (RR = 0.6). The age of spaying was known for 15 cats, four of which were spayed before the age of 2 years. The study included an unusually high number of Siamese cats – 52 out of 110 – which could lead to sample bias. It is unclear from the article how cases were selected for the study and what primary data were used for relative risk calculations.

A case-control study (Overley et al., 2005) evaluated the influence of age of ovariohysterectomy, parity, and progestin exposure on the risk of feline mammary carcinoma development. Overall, intact female cats had a significantly higher risk of developing mammary carcinoma (OR 2.7). Stratification by spay age revealed that female cats had a 91% reduction in the risk of developing a feline mammary carcinoma if spayed prior to 6 months of age and an 86% reduction in risk if spayed prior to 1 year of age compared with intact female cats. Ovariohysterectomy performed after two years of age increased the risk of feline mammary carcinoma development when compared with intact female cats. The study authors explain this result by the small number of cats spayed after 2 years of age. Additionally, it is possible that cats older than 2 years were spayed due to the development of mammary tumours. This study has many limitations. Although data were collected on potential confounding factors (parity, progestin exposure), no multivariate analysis was performed to account for the degree of influence of each. Spay age and other reproductive history data were collected using questionnaires sent to owners or referring veterinarians. But the questions were about long-past events and there is a risk of confusion. The control population included female cats diagnosed with either benign or malignant tumours of organs other than the mammary gland. Because spaying can be protective of mammary tumours but can be increasing the risk of other tumours, the use of cats with benign or malignant tumours as controls is a confounding factor. Another potential source of bias is that the number of intact cats may reflect the propensity of owners not to seek veterinary care unless absolutely necessary, therefore entry into the biopsy service may not reflect the prevalence of mammary tumours in intact cats, but the behaviour of the owners.

A large case-control study (Graf et al., 2016) analysed the influence of sex, neutering status, breed, and age on the development of the most common feline tumour types and tumour locations. The data are based on analysis of the Swiss Feline Cancer Registry, which consists of 51322 feline patient records compiled between 1965 and 2008. After removal of duplicate entries and incomplete data, 41045 individual cats were included in the analysis. Regarding the PICO question, there were 1501 female cats with mammary tumours. Analysis revealed that the odds of spayed female cats developing a mammary tumour compared with entire female cats were significantly lower (OR 0.62 for all mammary tumours and OR 0.69 for malignant mammary tumours). The data were collected retrospectively over several decades, so there is a risk of bias, and the results can only be generalised to those animals, the data for which were included in the cancer registry. This study did not take the age of spaying into account, and it is not clear how the data were collected. Additionally, although it not directly relevant to the PICO question at hand, this study made an important observation: spayed female cats had significantly higher odds of developing fibrosarcoma, lymphoma, squamous cell carcinoma than entire female cats. These results demonstrate that it is incorrect to assess the impact of spaying alone one aspect of female cats health – it may reduce the risk of mammary tumours but increase the risk of other tumours.

In addition to those described above, the search identified another case-control study (Misdorp et al., 1991) investigating the effect spaying on mammary tumours risk in female cats. According to the available abstract, ovariectomy was found to protect against mammary carcinomas but not against benign mammary tumours. However, the full text of this study could not be found, so it was not possible to critically evaluate it.

Case-control and cohort studies are in the middle of the evidence pyramid and can be good designs for risk assessment if designed correctly. Existing studies have examined PICO question as part of larger studies, so some data are limited. However, these studies provided some potentially important results that require further study. Thus, Overley et al. (2005) found an increased risk of developing mammary tumours in cats spayed over the age of 2 years, and according to Graf et al. (2016) spayed cats have an increased risk of developing other tumours. These data also carry the risk of bias, but they may cast doubt on the safety of spaying cats and show that it is incorrect to look solely at the risk of mammary tumour.

However, cats are often spayed not to reduce the risk of mammary tumours, but because of unacceptable behaviour and reproductive control. Considering the data from Overley et al. (2005) on the effects of endogenous progesterone and oestrogen on the risk of developing mammary tumours, the number of oestrous cycles – or age, since cycles are difficult to count in cats – before spaying is critical. Therefore, evidence-based recommendations are relevant not so much for spaying itself, but for the age of its implementation.

In conclusion, despite the generally accepted recommendation to spay female cats to reduce the risk of mammary tumours, the available evidence provides weak support for it. Although the appraised studies do not contradict biological plausibility, they also do not provide convincing evidence.

Prospective, well-designed cohort studies with controls for potentially confounding factors can provide evidence of the effect of spaying and age at spaying on feline mammary tumours risk. Additionally, a Knowledge Summary examining the evidence for the effect of spaying on the lifespan of cats in general would be useful.

## Methodology

**Search Strategy:** 

Databases searched and dates covered:	CAB Abstracts on the OVID interface 1973 to 2024 Week 25 PubMed accessed via the NCBI website 1910 to June 2024
Search strategy:	CAB Abstracts: (cat or cats or feline or felines or felis).mp. or exp cats/ or exp felis/ (spey* or spay* or neuter* or ovariectom* or ovariohysterectom* or ovario-hysterectom* or hysterectom* or sterilis* or steriliz* or desex* or de-sex* or gonadect*).mp. or exp ovariectomy/ or exp sterilization/ or exp hysterectomy/ or exp gonadectomy/ ((mammar* or breast*) and (tumor* or tumour* or neoplas* or cancer* or carcinom* or adenocarcinom* or adenoma*)).mp. exp mammary gland neoplasms/ 1 and 2 and (3 or 4) PubMed: cat or cats or feline or felines or felis spey* or spay* or neuter* or ovariectom* or ovariohysterectom* or ovario-hysterectom* or hysterectom* or sterilis* or steriliz* or desex* or de-sex* or gonadect* (mammar* or breast*) and (tumor* or tumour* or neoplas* or cancer* or carcinom* or adenocarcinom* or adenoma*) 1 and 2 and 3
Dates searches performed:	25 Jun 2024

**Exclusion / Inclusion Criteria:** 

Exclusion:	Non-English language publications. Conference paper or thesis or book chapters. Case reports. Narrative reviews.
Inclusion:	Articles (primary research and systematic reviews) that assessed the risk of mammary tumours in cats based on spaying status, even if this was not the focus of the study.

**Search Outcome:** 

Database	Number of results	Excluded – non-English language	Excluded – conference paper or thesis or book chapters	Excluded – case reports	Excluded – narrative reviews	Excluded – not relevant to PICO	Excluded – full text not available	Total relevant papers
CAB Abstracts	102	44	10	10	11	25	0	2
PubMed	46	3	0	6	3	31	1	2
Reviewer suggestion and citation tracking								2
Total relevant papers when duplicates removed								4

## Acknowledgements

I am grateful to Clare Boulton for her invaluable help in developing and running the search strategy. My sincere thanks go to William Smith for his careful reading, constructive questions, and for allowing extra time when needed. I also thank the anonymous reviewers and Louise Buckley for their many helpful suggestions. Everyone I worked with was unfailingly friendly, polite, and genuinely engaged in improving this article.

## ORCiD

Elena Gogua: https://orcid.org/0000-0001-8186-5759

## Conflict of Interest

The author declares no conflicts of interest.
